# COVID-19 Death Rates in Iran and Iraq: Possible Relations Between Iraq’s Pre-COVID-19 Mass Gatherings and Its Low Death Rate

**DOI:** 10.1007/s44197-022-00072-2

**Published:** 2022-10-21

**Authors:** Amir Amani, Hasan Namdar Ahmadabad, Abbas Nikravesh, Javad Zarei, Ali Haghbin

**Affiliations:** 1 Natural Products and Medicinal Plants Research Center, North Khorasan University of Medical Sciences, Bojnurd, Iran; 2 Department of Advanced Technologies, School of Medicine, North Khorasan University of Medical Sciences, Bojnurd, Iran; 3 Department of Pathobiology and Laboratory Sciences, School of Medicine, Vector-Borne Diseases Research Center, North Khorasan University of Medical Sciences, Bojnurd, Iran; 4 Department of Health Information Technology, School of Allied Medical Sciences, Ahvaz Jundishapur University of Medical Sciences, Ahvaz, Iran; 5 Department of Pediatrics, School of Medicine, North Khorasan University of Medical Sciences, Bojnurd, Iran

**Keywords:** COVID-19, Death rates, Iran, Iraq, Mass gathering

## Abstract

In recent years, COVD-19 has made millions of death worldwide. When reviewing the death rate, we encountered a very notable difference in death rate of Iran and Iraq, which are two neighboring countries. Investigating the COVID-19 risk factors, parameters, such as ethnicity and vaccination, do not appear not to be affecting our observation. We also could not find important differences in mortality rate being under-reported in the two countries. In this letter, we tried to discuss the possible effect of Iraq pre-COVID-19 mass gatherings on the death rate. The authors would like to highlight the effect of immune system on COVID-19.

## Introduction

COVID-19 is caused by severe acute respiratory syndrome coronavirus 2 (SARS-CoV-2). The new respiratory disease has spread widely throughout the world during the last year and made important changes in the lifestyle across the world. About 6,400,000 deaths have been officially reported so far from this disease [1]. Although the disease is now affecting nearly all countries, an important variation in deaths has attracted our attention: Iran and Iraq are two neighboring countries. However, looking into the deaths reported, the deaths per 1 million population (1MP) in Iraq are substantially smaller than that of Iran (i.e., 601 vs. 1642) [1]. In this commentary, we are trying to discuss the possible reasons for this important difference. It is worth noticing that under-reporting the data, a possible reason for this observation, appears not to be notably different in the two countries [2]. Additionally, the number of corona tests in Iraq and Iran is more or less similar: tests per 1MP in Iraq are 439,822 and this value is 582,345 in Iran [1], another reason which can indicate that under-reporting should not be considerably different in the two countries.

## Main Text

Looking into the literature, various reasons have been reported as risk factors for the disease. Here, we try to provide a discussion of their possible effects on the mortality rate of the two countries. The authors appreciate that providing an extensive comparison is a very complicated task and many confounding variables may influence our assessment. However, to the best of our knowledge, an acceptable comparison has been provided here:

- Vaccination: when measuring all vaccine doses (including boosters), so far, total dose equal to about 172% of Iran's population has been injected in Iran, while this share is only 43% in Iraq [3].
- COVID-19 variant: Although we could not find strong evidences detailing dominant strains of COVID-19 as a function of time in the two countries; apparently, no important variation in the dominant strain may be detected [4].
- Management protocols: both countries have tried to follow the WHO suggested protocols for management of COVID-19 [4].
- Wearing face mask: we could not find data about number of people who wear face mask in Iraq during the last years. However, in Oct 2020, an Iraqi official declared that only 20% of Iraqis were wearing masks [5], while it was reported as 55.35% in Iran in a similar time period [6].
- Air pollution: based on the data provided by international association for medical assistance to travelers, the annual mean concentration of PM2.5 for Iran and Iraq is 39 and 62 µg/m^3^, respectively [7].
- Underlying diseases: from a report by WHO (World Health Organisation), 10.3% of Iranians and 13.2% of Iraqis [8] suffer from diabetes. Hypertention rate in Iran is also estimated to be considerably less than that of Iraq [9].
- Obesity, physical inactivity and smoking: prevalence of obesity in Iran (25.8% [10]) is lesser than that of Iraq (30.4% [10]). Also, based on the report by WHO, physical inactivity in Iran (31.9%) is considerably lesser than that of Iraq (46.3% [8]). Furthermore, comparison of tobacco users in Iran (14.1%) and Iraq (19.6%) shows less smokers in Iran [11].
- Ethnicity: in our case, due to the linguistic, religious, and geographic commonalities of the two neighboring countries as well as economic, commercial, cultural, and religious exchanges throughout the history, we cannot predict notable variation in ethnicity of Iranians and Iraqis. Additionally, from COVID-19 registry system in Ahvaz University of Medical Sciences (i.e., Khuzestan province of Iran which is bordering Iraq), by 22-Aug-2021, the death rate per 1MP was 1466 [12], relatively larger than that of other provinces of Iran. Considering the similar ethnicity of Khuzestanis (Iran) and Arabs of Iraq, the effect of ethnicity of the death rate appears not to be important.
- Age: looking into the demographic information of the two countries, Iraq is a substantially younger country (median age 21.2 years) in comparison with Iran (median age 31.7 years)—percentage of people above 55 in Iraq is also considerably less than that of Iran (i.e., 7.56% vs. 13.59%, respectively) [10].

Comparing the factors mentioned above, the only risk factor which we could find favoring the lesser death rate in Iraq is age. Although age is a very important risk factor, by studying the age in other countries, this single factor cannot address the huge difference between the mortality rate of Iran and Iraq. For instance, Kuwait, an Arab country with median age 29.7 years, has death rate close to Iraq (ie. 581 per 1MP [1]). On the other hand, in Bolivia (median age 25.3 years), the death rate is even more than that of Iran (1833 per 1MP [1]).

We therefore believe that other variables should be considered in this case. Looking into the social behavior of Iraqis, several mass gatherings throughout the year are being held in Iraq annually: the most important one is “Arbaeenia”. More than 20 million people (i.e., around half of Iraq's population) gathered in Karbala city in 2016, of which about 20% came from other countries [13]. This number increased every year up to 2019. Throughout the year, some other gatherings, involving more than 1 million people, are also observed in Iraq too, while such gatherings have been rarely held in Iran in recent decade. We know that during such gatherings, respiratory tract infections spread quickly between the people [14]. It is arguable that such infections act as stimulants for immune system. From hygiene hypothesis, we already know that improper development of immune system is expected wherever people follow hyper-hygienic regimens [15]. In a country like Iran, with higher hygiene levels, the population is not expected to receive the above-mentioned stimulants for immune system. Therefore, Iranians may not be “naturally” immunized against new pathogens. Consequently, when encountering the new pathogens, immune disorders, such as hyperinflammation, could be anticipated.

The severe form of COVID-19 is developed following excessive inflammatory responses and cytokine storm. Dysregulation of immune system through alleviation of T cells, increasing of IL-6 level, as well as reduction of B cells and natural killer (NK) cells has been reviewed in COVID-19 [16]. The authors believe that the developed immune system of Iraqis (due to mass gatherings) may play an important role in low death rate from COVID-19 in this country. Our idea has been well explained by Sehrawat and rouse [17]. In brief, considering the hygiene hypothesis, they address the issue though the trained immunity: repeated exposure to the pathogens activates the innate immunity for many months. In this type of immunity, when the response is generated against one set of microbes, protection against other infections is expected. Such bystander immunity is from different immune cells, e.g., macrophages, NK cells, innate lymphocytes, and dendritic cells [18]. We therefore suggest that during the pre-COVID-19 mass gatherings in Iraq (which continued during the COVID-19 pandemic but with smaller number of participants), the immune system of Iraqis has developed against new respiratory infections. Hence, lesse mortality has been observed during the COVID-19 pandemic in Iraq.

A second effect from the mass gatherings in Iraq is the change in the microbiome profile of the Iraqis. The microbiome may play critical role in the training and development of major components of immune system [19]. One may therefore expect improved reaction to COVID-19 virus in them.

To conclude, this commentary argues that the Iraqis might have become more resistant to SARS-CoV-2 through their social behavior, especially mass gatherings. At this point, anticipating future viral pandemics, we would like to raise the question by Ghanaemi and Yoshioka that are we "losing our immunity when we need it the most" [20]?

## Data Availability

Not applicable.

## Abbreviations

1MP: 1 Million population
WHO: World Health Organisation

## References

[1] Worldometers.info. COVID-19 coronavirus pandemic. Access Date: 20 July 2022. Available from: https://www.worldometers.info/coronavirus/.

[2] Wang H, Paulson KR, Pease SA, Watson S, Comfort H, Zheng P, Aravkin AY, Bisignano C, Barber RM, Alam T, Fulle JE, May EA, Jones DP, Frisch ME, Abbafati C, Adolph C, Allorant A, Amlag JO, Bang-Jensen B, Bertolacci GJ, Bloom SS, Carter A, Castro E, Chakrabarti S, Chattopadhyay J, Cogen RM, Collins JK, Cooperrider K, Dai X, Dangel WJ, Daoud F, Dapper C, Deen A, Duncan BB, Erickson M, Ewald SB, Fedosseeva T, Ferrari AJ, Frostad JJ, Fullman N, Gallagher J, Gamkrelidze A, Guo G, He J, Helak M, Henry NJ, Hulland EN, Huntley BM, Kereselidze M (2022). Estimating excess mortality due to the COVID-19 pandemic: a systematic analysis of COVID-19-related mortality, 2020–21. Lancet.

[3] Ourworldindata.org. Coronavirus (COVID-19) Vaccinations. Access Date: 20 July 2022. Available from: https://ourworldindata.org/covid-vaccinations?country=OWID_WRL.

[4] Al-Lami F. Personal Communication; 2022.

[5] Jawad A. Only 1 of 5 Iraqis 'committed to wearing masks'. www.aa.com; 2020.

[6] ISNA. The downward trend of health protocols and the re-ascent of the corona in the country; 2020.

[7] IAMAT. Genral health risks: air pollution; 2021.

[8] WHO. Diabetes country profiles; 2016.

[9] Kirschbaum TK, Theilmann M, Sudharsanan N, Manne-Goehler J, Lemp JM, De Neve JW, Marcus ME, Ebert C, Chen S, Aryal KK, Bahendeka SK, Norov B, Damasceno A, Dorobantu M, Farzadfar F, Fattahi N, Gurung MS, Guwatudde D, Labadarios D, Lunet N, Rayzan E, Moghaddam SS, Webster J, Davies JI, Atun R, Vollmer S, Bärnighausen T, Jaacks LM, Geldsetzer P (2021). Targeting hypertension screening in low-and middle-income countries: a cross-sectional analysis of 1.2 million adults in 56 countries. J Am Heart Assoc.

[10] Index Mundi. Country comparisons; 2021.

[11] Sohrabi MR, Abbasi-Kangevari M, Kolahi AA (2020). Current tobacco smoking prevalence among Iranian population: a closer look at the STEPS surveys. Front Public Health.

[12] Zarei J, Dastoorpoor M, Jamshidnezhad A, Cheraghi M, Sheikhtaheri A (2021). Regional COVID-19 registry in Khuzestan, Iran: a study protocol and lessons learned from a pilot implementation. Inform Med Unlocked.

[13] Al NM (2020). Iraq mass gathering preparedness and public health recommendations. JMIR Public Health Surv.

[14] Petersen E, Memish ZA, Zumla A, Maani AA (2020). Transmission of respiratory tract infections at mass gathering events. Curr Opin Pulm Med.

[15] Preidis GA, Versalovic J (2009). Targeting the human microbiome with antibiotics, probiotics, and prebiotics: gastroenterology enters the metagenomics era. Gastroenterology.

[16] Tufan A, Avanoğlu Güler A, Matucci-Cerinic M (2020). COVID-19, immune system response, hyperinflammation and repurposing antirheumatic drugs. Turk J Med Sci.

[17] Sehrawat S, Rouse BT (2020). Opinion: Does the hygiene hypothesis apply to COVID-19 susceptibility?. Microbes Infect.

[18] Netea MG, Joosten LA, Latz E, Mills KH, Natoli G, Stunnenberg HG, O’Neill LA, Xavier RJ (2016). Trained immunity: a program of innate immune memory in health and disease. Science.

[19] Zheng D, Liwinski T, Elinav E (2020). Interaction between microbiota and immunity in health and disease. Cell Res.

[20] Ghanemi A, Yoshioka M, St-Amand J (2021). Coronavirus disease 2019 (COVID-19) crisis: losing our immunity when we need it the most. Biology.

